# Ex vivo models for intestinal translocation studies of cellulose nanocrystals

**DOI:** 10.1007/s44164-023-00056-x

**Published:** 2023-08-21

**Authors:** Michelle Müller, Roland Drexel, Marie Burkhart, Stephan Dähnhardt-Pfeiffer, Lena Wien, Christine Herrmann, Thorsten Knoll, Christoph Metzger, Heiko Briesen, Sylvia Wagner, Florian Meier, Yvonne Kohl

**Affiliations:** 1 Department Bioprocessing & Bioanalytics, Fraunhofer Institute for Biomedical Engineering IBMT, Joseph-von-Fraunhofer-Weg 1, 66280 Sulzbach, Germany; 2 Postnova Analytics GmbH, Rankinestr. 1, 86899 Landsberg am Lech, Germany; 3 Microscopy Services Dähnhardt GmbH, Plambeckskamp 2, 24220 Flintbek, Germany; 4 Process Systems Engineering, School of Life Sciences, Technical University Munich, Gregor-Mendel-Str. 4, 85354 Freising, Germany

**Keywords:** Mucus barrier, Microfluidics, Primary intestinal tissue, Asymmetrical flow field flow fractionation, Electron microscopic imaging

## Abstract

**Purpose:**

Cellulose nanocrystals (CNC) play a promising role in the development of new advanced materials. The growing demand of CNC-containing products in the food industry will lead to an increased human exposure through oral uptake. To date, there is a dearth of studies reporting on the risks which CNC pose to human health following ingestion. In vitro models, which lack physiological accuracy, are often used to justify animal experiments in the field of nanosafety assessment. Nevertheless, ex vivo models of the intestine pose promising alternatives to in vivo experiments.

**Methods:**

Two ex vivo models, a microfluidic chip based on porcine intestinal mucus and the Ussing chamber apparatus with tissue from abattoirs, which aim to complement in vitro models, are characterized by investigating the transport and toxicity of CNC through them in comparison to an in vitro triple co-culture model. Silver nanoparticles were included in this study as well-known and characterized nanomaterials for comparative purposes.

**Results:**

Study results show that CNC cross the intestinal mucus layer but do not pass the intestinal tissue barrier ex vivo and in vitro; furthermore, no toxic effects were observed under exposure conditions tested.

**Conclusion:**

These ex vivo models present complementary methods to the existing standardized in vitro and in silico methods to support data generation under physiologically relevant conditions without the use of animals. This multi-model approach offers an enhanced understanding of the complex interaction between new materials and human tissue and aligns with the flexible approach of IATA (Integrated Approaches to Testing and Assessment) and NAMs (New Approach Methods) for chemical and drug safety assessment.

**Supplementary Information:**

The online version contains supplementary material available at 10.1007/s44164-023-00056-x.

## Introduction

Cellulose is the most abundant natural occurring polymer worldwide and considered an inexhaustible source of environmentally friendly raw material for new and emerging products [1]. Traditionally, cellulose is used for the production of paper, cardboard, wood-derived products, and textile fibers [2]. Cellulose-derived or cellulose-reinforced biopolymers, especially cellulose nanocrystals (CNC), play a promising role in the development of new advanced materials. CNC are typically extracted from the natural cellulose contained in trees and plants and form rigid, rod-like particles of several nanometers in width and range from few to several hundred nanometers in length [3]. Their uniquely high mechanical strength in combination with optical absorption, barrier properties, and antimicrobial effects makes them well suited to future technologies such as those in the food sector [1, 4–6]. The increased development of CNC-containing food additive and food packaging products will inevitably lead to increased human exposure by oral ingestion. It has already been shown that conventional cellulose is not absorbed from the gastrointestinal tract (GIT), but this could be different for nanosized cellulose such as CNC, since they have different physicochemical properties [7]. After oral uptake, if CNC cross the mucus layer of the GIT, which poses the first absorption hurdle, it may also cross the intestinal barrier. Previous studies on pathogens [8, 9] and other drugs [10, 11] have shown that they are unable to pass through the mucus layer due to mucoadhesion. However, smaller and more negatively charged CNC may pass the mucus layer more easily [7]. Based on a small number of research studies, it is assumed that CNC absorption is negligible in vivo due to particle agglomeration and mucoadhesion in the intestine [12]. Further toxicological investigation is needed to improve the understanding of the adverse health effects from absorption and toxicity of CNC in the human GIT.

The 3R principle of Russell and Burch [13] encourages to reduce, replace, and refine animal testing whenever possible. However, this philosophy is currently not suited to in vivo experiments which demonstrate CNC safety for humans. The guidance of European Food Safety Authority (EFSA) [14] advises that rodent studies are performed alongside in vitro studies to demonstrate the safe use of novel food ingredients. Also in the case of chemical registration, the performance of various studies in rodents is currently mandatory to determine toxicity after oral ingestion (OECD Guidelines for the Testing of Chemicals Test No. 407 [15], 408 [16], 420 [17], 423 [18], 425 [19]). In vivo models are also used in biomedical research to reproduce human disease and develop new therapeutic options [20], but they poorly represent biological mechanisms which occur in humans. An increased understanding of these mechanisms on a molecular level, in combination with advanced technologies, is needed for the development and validation of non-animal methods (NAMs) for effective assessment in humans.

A battery of simple ex vivo methods, which mimic human gut physiology more closely than rodent models, are needed to complement in vitro experiments. Ex vivo models are considered a compromise between in vitro and in vivo models, since tissues extracted from organisms are used in a controlled external environment. Intestinal ex vivo models, like the everted gut sac model [21], the Ussing chamber method for isolated intestinal mucosae [22], and the rat or mouse intestinal loop/perfusion techniques [23], represent useful approaches to study the uptake of drugs in the intestine as alternatives to in vivo experiments [24]. However, none is currently accepted as alternative 3R methods. The EURL ECVAM DataBase Service on Alternative Methods to animal experimentation (DB-ALM catalogue distributed through EURL ECVAM collection by the JRC (Joint Research Centre of the European Commission)) [25] currently lists 371 methods that are accepted as alternatives which contribute to reducing and replacing animal experiments. Most offer alternatives to studies on eye irritation, skin irritation, and developmental toxicology and use in vitro and in silico techniques, as oppose to ex vivo.

Two reliable ex vivo approaches for the investigation of intestinal uptake of CNC are presented in this study: (A) a mucus chip model, which provides prediction and translocation of test substances and particles across the intestinal mucus layer, comprising the first hurdle to absorption and (B) a primary intestine barrier model based on an Ussing chamber system, which performs electrophysiological measurements to investigate the influence of CNC on the barrier properties of intact living tissue and the translocation of particles across the tissue. Since the understanding on the behavior of CNC in in vitro and ex vivo studies is very limited at present, silver nanoparticles are used in this study as an additional, well-known, and characterized nanomaterial for comparative purpose.

## Materials and methods

### Extraction of cellulose nanocrystals

Cellulose nanocrystals (CNC) were extracted from Whatman® ashless filter aids (WHA1703050, Merck KGaA) by sulfuric acid hydrolysis based on the protocols described by [26–28]. In brief, sulfuric acid (96 wt%, Carl Roth) was diluted to 62 wt% with deionized water (18.2 MΩ·cm, Milli-Q^®^ Direct 8 water purification system, Merck Chemicals) and pre-heated to 50 °C in a stirred tank reactor (Atlas, Syrris) equipped with an anchor-type stirrer. Filter papers (previously cut into small pieces and dried at 105 °C for 30 min) were then added at a ratio of 1/10 (cellulose/sulfuric acid, w/w). The reaction mixture was stirred constantly at 200 rpm for 70 min. The reaction was quenched by tenfold dilution with cold (4 °C) deionized water. Purification was obtained by twofold centrifugation (4594 rcf, 15 min, Centrifuge 5910 R, Eppendorf) and redispersion of the precipitate in deionized water followed by dialysis (ZelluTrans/ROTH T3 dialysis membrane, Carl Roth). Finally, the suspension was sonicated at a specific energy of 2 kJ·g^−1^ cellulose [29] and an amplitude setting of 30% (Sonopuls HD 3200 homogenizer equipped with a VS 70 T sonotrode, Bandelin) in an ice bath.

Transmission electron microscopic (TEM) analysis of negatively stained CNC was performed with a Philips CM10 instrument, coupled with a CCD camera (IDS, Obersulm), at an acceleration voltage of 80 kV. Negative staining and the complete preparation procedure of the CNCs was performed as reported in [2]. For in vitro and ex vivo studies, the CNC stock solution was mixed with pre-warmed (37 °C) cell culture medium (CCM) or Krebs-Ringer buffer (KRB) to create the test concentration of 100 μg·mL^−1^.

### Silver nanoparticles as reference nanomaterial

Silver nanoparticles (Ag-NP) with a mean diameter <20 nm (NM-300K, JRC) were obtained from the Fraunhofer Institute for Molecular Biology and Applied Ecology IME (Schmallenberg, Germany). The Ag-NP stock suspension was treated in an ultrasonic bath (Elmasonic S15, Elma) for 10 min to disrupt agglomerations, before mixing with pre-warmed (37 °C) cell culture medium (CCM) or Krebs-Ringer buffer (KRB) to create the test concentration of 1 mg·mL^−1^. Ag-NP are used in this study as a well-known and characterized nanomaterial for comparative purpose.

### Characterization of nanomaterials

#### Determination of surface charge density

The surface charge density (OSO^3−^) of the CNC was determined by conductometric titration (Konduktometer 703 with an electrode sensor SE 204, Knick) according to [30].

#### Dynamic light scattering

The hydrodynamic diameter of CNC was determined by dynamic light scattering using a Zetasizer Nano ZSP (Malvern Instruments, Worcestershire, UK) equipped with a red laser (633 nm) under a backscatter detection angle of 173° after the extraction. All samples were measured at 25 °C in triplicate and are reported as means with standard deviations obtained from the cumulant analysis.

#### ζ-Potential measurement

The ζ-potential of CNC and Ag-NP was measured by electrophoretic light scattering with a Zetasizer Nano ZSP (Malvern Instruments) using the Smoluchowski approximation. To determine the surface charge of CNC and Ag-NP in cell culture media, the protocol reported by [31] was used.

#### Characterization and detection of translocated particles by asymmetrical flow field flow fractionation (AF4)

Ultrapure water (UPW, resistance 18.2 MΩ∙cm) was derived from a Milli-Q system (Integral 5 system, Merck). The AF4 fractionation system (AF2000, MT, Postnova Analytics GmbH (PN), Landsberg am Lech, Germany) included an autosampler (PN5300), a slot-outlet (PN1650), and a channel thermostat (PN4020). The analytical fractionation channel with a tip-to-tip length of 277 mm was equipped with a regenerated cellulose membrane (RC) of 10 kDa molecular weight cut-off and a Mylar spacer of 350 μm height. The channel thermostat was used to stabilize the channel temperature at 25 °C, whereas the samples were stored in the autosampler at 6 °C. The fractionation system was hyphenated to a UV/Vis detector (PN3211) and a multi-angle light scattering detector (MALS, PN3621 with 21 angles). The absorbance was detected at 254 nm for all CNC samples and 420 nm for Ag-NP samples, respectively. The instrument control and data evaluation was performed using the NovaFFF software (Version 2.1.0.4, PN). To comprehensively investigate the studies involving Ag-NPs, the fractionated samples were further analyzed online using an ICP-MS instrument (ICP-MS 7900, Agilent Technologies) as an elemental detector. Therefore, the AF4 was directly hyphenated to the ICP-MS instrument, where the isotopes ^107^Ag and ^103^Rh were monitored. The integration time for the silver isotope ^107^Ag was set to 0.5 s. A helium flow rate of 4.5 mL·min^−1^ was used as a collision gas to remove potential interferences. The peristaltic pump of the ICP-MS instrument introduced a 10 ppb solution of Rh as an internal standard in 1% nitric acid. The sample introduction system and the plasma conditions were identical to the spICP-MS experiments. The AF4 measurement conditions and parameters for CNC fractionation were reported recently [28]. After filtration of UPW through a 0.1 μm pore size membrane (Durapore, Merck Millipore), the carrier liquid was prepared by adjusting the pH to around 9.4 using 0.1 M sodium hydroxide (Carl Roth GmbH & Co. KG) for the fractionation of Ag-NP. The fractionation method for Ag-NP consisted of a 6 min injection and focussing phase using an injection flow of 0.2 mL·min^−1^ and an initial cross flow rate of 1.2 mL·min^−1^. A detector flow rate of 0.3 mL·min^−1^ with additional 0.2 mL·min^−1^ for the slot outlet was applied. Firstly, the elution used a constant 2 min step followed by a 40 min long linear decay down to a cross flow rate of 0.1 mL·min^−1^, which was kept constant for additional 20 min. A 15 min rinse step at the end was implemented to remove potential larger aggregates and to minimize potential carry-over effects. Due to inhomogeneities and the presence of precipitate in the ex vivo and mucus samples originating from the complex biological samples, all samples were filtered through 1.0 μm glass fiber syringe filters (Chromafil Xtra, Machery-Nagel) prior to injection into the AF4.

#### Determination of size distribution and ionic concentration via single particle inductively coupled plasma mass spectroscopy (spICP-MS)

spICP-MS experiments for the detection and characterization of Ag-NPs were carried out on an Agilent ICP-MS 7900 (Agilent Technologies). The calibration of the analyte and reference materials and the determination of the nebulization efficiency were performed using certified ionic silver and gold standards and a reference standard material of gold nanoparticles (Au-NP) with a nominal diameter of 58 nm (product code EM.GC60, BBI Solutions, Crumlin, UK), respectively. The instrument tune and calibration were conducted on a daily basis. A detailed description of the procedure and the measurement conditions is given in a previous work [32]. All samples were diluted with UPW to obtain optimal concentrations for spICP-MS measurements. spICP-MS experiments were conducted to determine the size distribution of potentially translocated particles due to its high sensitivity and low detection limit for Ag-NP.

### Porcine intestinal tissue and mucus preparation

For intestinal mucus and tissue preparation, fresh small intestine from *Sus scrofa domestica* was provided by a local slaughterhouse in Zweibruecken, Germany. The animals were slaughtered and cut up referred to Council Directive 93/119/EC of the European Commission [33] on the protection of animals at the time of slaughter or killing, dated December 22, 1993. The fresh tissue was divided into pieces and rinsed with phosphate-buffered saline (PBS). Then it was opened along the mesenteric line and laid down with the mucosal side faced up. Mucus was gently isolated with a Teflon spatula and transferred into a tube. The isolated primary porcine small intestinal mucus was stored at 4 °C and used within 48 h after preparation. To ensure repeatable properties of the primary mucus, rheological measurements were performed after each tissue preparation [34]. For Ussing chamber experiments, the opened intestine was cut into 2 cm × 2 cm slices and mounted with the mucosal side faced down on tissue inserts with a test surface of 0.5 cm^2^, which were afterwards transferred to the Ussing chamber apparatus. To ensure consistent properties, only tissue with an electrical resistance >20 Ohm cm^−2^ was used within 12 h after slaughtering.

### Characterization of intestinal tissue via electron microscopy

For electron microscopy investigation of the intestinal tissue, slices were chemically fixed, dehydrated, and embedded in resin and sectioned as described previously [35]. Transmission electron microscopical investigation was carried out by a Philips TEM CM10 (FEI) at 80 kV. Images were captured with a CCD camera (μ-Eye, IDS Imaging) connected directly to the TEM. For scanning electron microscopy, the fixed and dehydrated tissue was dried by critical point drying using a CPD 010 (Baltec). After drying, the samples were mounted on aluminum stubs (Plano) and coated with gold using a sputter coater SCU 030 (Baltec). The investigations with the SEM DSM 940 (Zeiss) were performed with 10 kV, and the images were captured using the software DISS 5 (point electronic).

### Translocation studies with porcine mucus (microfluidic mucus barrier model)

The fabrication and composition of the mucus chip module as well as mucus preparation, characterization, and performance of permeation study is described in detail in [34]. Briefly, the mucus chip system consists of a microfluidic cartridge and an insert with the porcine mucus, which is connected with peripheral fluidic components. The cartridge has one open sample compartment on the apical side, where the test substances were applied directly onto the mucus layer. A microfluidic channel on the basolateral side was used for delivery of the permeated samples to a collecting reservoir for analysis. The chip setup was used to study the permeation of the test compounds caffeine (Sigma-Aldrich), FITC-dextran 70 kDa (Sigma-Aldrich), Ag-NP, and CNC over the mucus membrane under fluidic conditions. The mucus was filled into a metal grid with a thickness of 200 μm to mimic the thickness of the small intestine in vivo. The microfluidic setup was placed inside an incubator with a temperature of 37 °C. Krebs-Ringer buffer (KRB) (pH 7.3) was pumped through the lower channel by a peristaltic pump (Reglo Digital MS-4/12–100, Cole-Parmer) with a speed of 0.167 mL·min^−1^ as acceptor fluid. About 200 μL of the sample solution was placed on the apical side with a concentration of either 100 μM caffeine (Sigma-Aldrich), 100 μM 70 kDa FITC-dextran (Sigma-Aldrich), 100 μg·mL^−1^ CNC, or 1 mg·mL^−1^ Ag-NP. Samples were taken after 2 h. Permeated amount of caffeine was analyzed by high performance liquid chromatography (HPLC) as described in detail in [34]. Briefly, the analysis was performed with an Agilent 1260 Infinity Quaternary liquid chromatography (LC) System (Agilent Technologies), equipped with a diode array detector (DAD G1315D, Agilent Technologies) having a detection wavelength of 275 nm. About 5 μL of the sample was injected and the elution was performed with a gradient of water (solvent A), acetonitrile (solvent B), and 0.1% trifluoracetic acid in water (solvent C) at a flow rate of 0.4 mL·min^−1^. A Poroshell 120 EC-C18 column (2.1 × 100 mm, 2.7 μm; 695775-902, Agilent Technologies) with a guard-column (Poroshell 120 EC-C18, 2.7 μm, 2.1 × 5 mm, Agilent Technologies) was used. The fluorescence of the permeated FITC was measured using a Tecan Reader Infinite F200 plate reader (Tecan Group Ltd.) with excitation and emission wavelengths of 485 nm and 535 nm. Permeated CNC and Ag-NP were subsequently analyzed by AF4 or spICP-MS, respectively.

### Transport and tissue integrity with intestinal ex vivo model (Ussing chamber)

To test for the translocation of CNC and the influence on the integrity of intestinal tissue ex vivo, the Easy Mount 4-chamber system (Physiologic Instruments) was used. Agar-agar (Sigma-Aldrich) dissolved in 3 M potassium chloride (Sigma-Aldrich) and solidified in electrode holders, served as electrical bridge between KRB and the voltage and current electrode pairs. Before the measurement, the apparatus was pre-heated to 37 °C and chambers were flushed with carbogen gas (95% O_2_, 5% CO_2_, Linde AG). First, measurements with KRB without tissue and supplements were performed to determine the electrode offset. Intestinal slices with a tissue area of 0.5 cm^2^ were mounted in the brackets and inserted between the chambers. About 5 mL of pre-warmed KRB supplemented with 10 mM glucose as energy substrate was added into the serosal chamber, and 5 mL pre-warmed KRB supplemented with 10 mM mannitol was added into the mucosal chamber to stabilize pressure. Measurements were performed in “open circuit” mode, which determines the difference of potential between the voltage electrodes after applying short current impulses and allows for the calculation of transepithelial electrical resistance (TEER) by Ohm’s law. After an equilibration period of 10 min, test substances were applied in the mucosal (apical) chamber. After 2 h, samples were taken on the serosal (basolateral) side and analyzed for translocated CNC or Ag-NP by AF4 or spICP-MS.

### In vitro triple co-culture model

Caco-2, a human adenocarcinoma cell line with epithelial morphology, and THP-1, a human monocytic cell line derived from an acute monocytic leukemia patient, were obtained from DSMZ (Deutsche Sammlung für Mikroorganismen und Zellkulturen GmbH). HT29-MTX-E12, a mucus-secreting subclone from colon adenocarcinoma HT29 cells, differentiated into mature goblet cells by methotrexate, was obtained from Sigma-Aldrich (product no. 12040401). All cell lines were cultured in Dulbecco’s modified Eagle’s medium (DMEM) high glucose (4.5 g·L^−1^) (Invitrogen) supplemented with 10% fetal calve serum (FCS) (Invitrogen), 2 mM L-glutamine (Invitrogen), 1% penicillin/streptomycin (Invitrogen), and 1% non-essential amino acids (Invitrogen). All cell lines were cultured in a humidified incubator at 37 °C and 5% CO_2_ and passaged twice a week. Twenty-four hours prior to the seeding for the translocation and toxicity studies, THP-1 cells were differentiated to adherent macrophage-like cells at a cell density of 4.0 × 10^5^ cells mL^−1^ in CCM supplemented with 20 ng·mL^−1^ PMA (Phorbol-12-myristat-13-acetat, Sigma-Aldrich). For the in vitro viability studies, 21-day differentiated triple co-cultures consisting of Caco-2, HT29-MTX-E12, and THP-1 in Transwell^®^ inserts with a pore size of 3.0 μm and a growth area of 1.12 cm^2^ (Corning) were used and prepared as described in [32]. Briefly, 2.5 × 10^5^ differentiated THP-1 cells were seeded basolaterally on flipped Transwell^®^ inserts and cultured for 1 h at 37 °C. Caco-2 and HT29-MTX were added at a density of 1.0 × 10^5^ per insert (ratio 9:1) apically (on the back flipped inserts) and cultured for 21 days.

### Transport and toxicity studies with intestinal in vitro model

Transport and toxicity studies were performed as described previously [34]. Cell culture medium of the triple co-culture model was removed and replaced by 500 μL test substance containing solutions. The cells were exposed at 37 °C for 2 h up to 24 h. Cell viability of the triple co-culture model was determined using the alamarBlue™ assay (Invitrogen), according to the manufacturer’s instructions. After incubation time, supernatants were discarded and alamarBlue™ reagent (10% solution in cell culture medium) was added apically (500 μL). Empty cell culture inserts without cells served as blank controls. 1% TritonX-100 in cell culture medium was applied as cell damaging positive control. The cells were incubated for 1 h at 37 °C. Afterwards, 100 μL of the apical supernatant was transferred from each cell culture insert into a minimum of three wells of a black 96-well plate (Greiner Bio-One) for fluorescence measurement using a Tecan Infinite F200 plate reader (Tecan) at an excitation/emission wavelength of 560/610 nm. Data evaluation was performed on Tecan i-control software (Version 1.9.17.0, Tecan). The data calculation was performed in Excel (Microsoft Office 2016).

### Statistical analysis

All data in this study are presented as mean values ± standard deviation (SD) from three independent experiments. The data were tested for statistical significance with the unpaired *t*-test in the GraphPad Online software 2022.

## Results

### Characterization of cellulose nanocrystals and silver nanoparticles

**Fig. 1:**
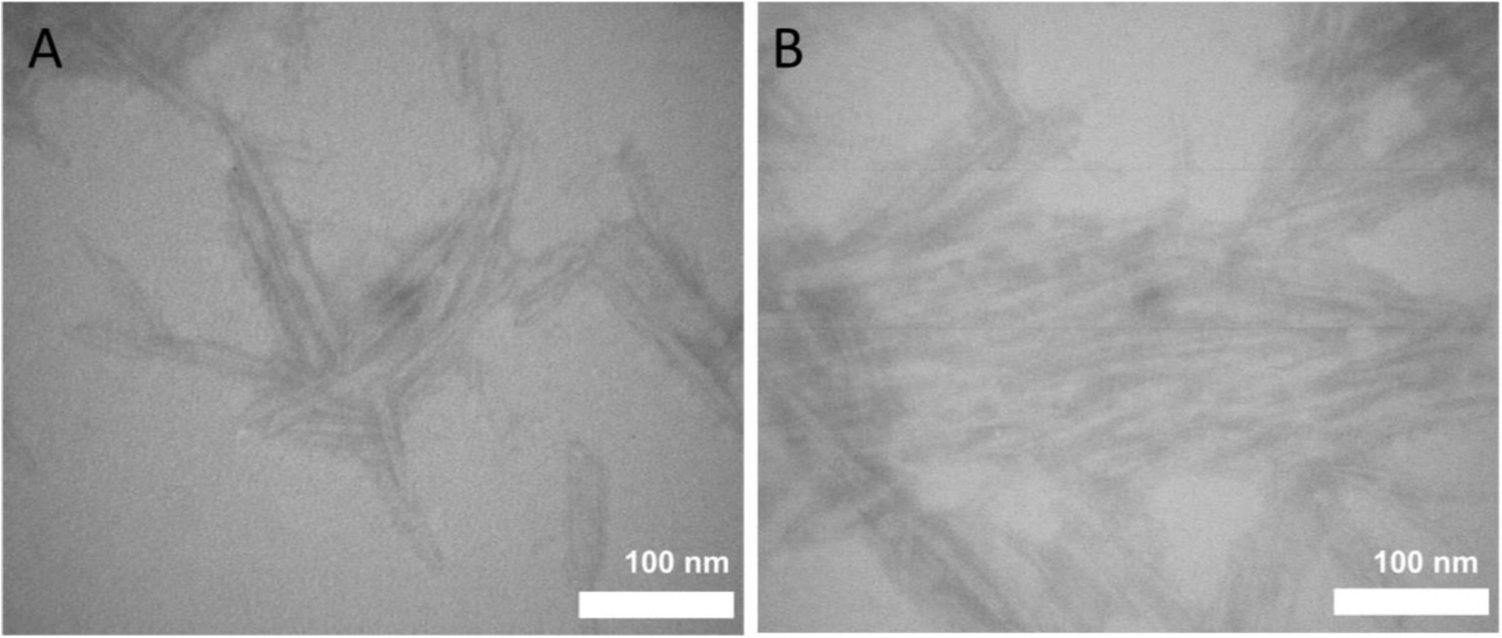
Transmission electron microscopy images of negatively stained cellulose nanocrystals. Single fiber molecules as well as agglomerated fibrous structures can be identified. **A** Small cluster of cellulose nanocrystals. **B** Large bundles of cellulose nanocrystals

The hydrodynamic diameter was determined as 52.3 ± 6.4 nm for the extracted cellulose nanocrystals (CNC) and 47.2 ± 1.0 nm for the Ag-NP. Nanomaterials were characterized in Krebs-Ringer buffer (KRB) and cell culture medium (CCM) to determine the stability of the suspension and potential agglomeration. AF4-MALS revealed slight agglomeration of CNC in KRB and CCM. Compared to CNC in ultrapure water, the radius of gyration (Rg) at the 90° MALS angle signal intensity maximum shifted to larger sizes for CNC in both KRB and CCM; at the same time, the peak maximum shifted to later retention times, and an increased peak width was observed (Fig. S1). In AF4, later retention times correlate with larger hydrodynamic sizes and the peak width is a measure for the broadness of the size distribution. The ζ-potential indicated a negative surface charge of CNC in both KRB and CCM (Table 1). Transmission electron microscopy (TEM) images revealed the fiber structure of CNC, which are forming bundles and sometimes larger agglomerates (Fig. 1). The mean particle size of the silver nanoparticles (Ag-NP) was determined in KRB to 32.2 ± 1.0 nm indicating slight agglomeration and in CCM to 15.0 ± 1.0 nm. (Fig. S2). The ζ-potential indicated a slightly negative surface charge of Ag-NP in both KRB and CCM (Table 1). MALS evaluations are not able to access the Ag-NP particles’ size because of surface plasmon resonance.

**Table 1:** Physicochemical characteristics of cellulose nanocrystals and silver nanoparticles in the used media

Size (nm)/technique	CNC		Ag-NP	
	KRB	CCM	KRB	CCM
R_g_ (90° MALS angle intensity maximum)/AF4-MALS	83.5 ± 2.2	95.5 ± 2.9	/	/
mass-based diameter/spICP-MS	/	/	32.2 ± 1.0	15.0 ± 1.0
ζ-Potential (mV)	−21.3 ± 0.9	−14.9 ± 0.1	−11.9 ± 0.5	−15.0 ± 1.0

*R*_*g*_ radius of gyration, *CNC* cellulose nanocrystals, *Ag-NP* silver nanoparticles, *KRB* Krebs-Ringer buffer, *CCM* cell culture medium

### Characterization of primary intestinal mucus

Porcine mucus samples have natural variations, based on their water content. Rheology was performed on mucus samples prior to use, to ensure that the viscosity is consistent between tests. According to a previously published study [33], the viscosity should be maintained between 30 and 45 Pa·s to be comparable with the properties of human GIT mucus [36]. The mucus chip experiments presented in this study were performed with mucus originating from two different batches. Batch 1 had a viscosity of 45.16 ± 1.49 Pa·s and a pH value of 5.75. Batch 2 had a viscosity of 32.18 ± 0.77 Pa·s and a pH value of 6.52. Both batches were within the accepted range, ensuring that repeatable mucus properties were used for the translocation studies of CNC and Ag-NP in the mucus chip.

### Characterization of primary intestinal tissue

**Fig. 2:**
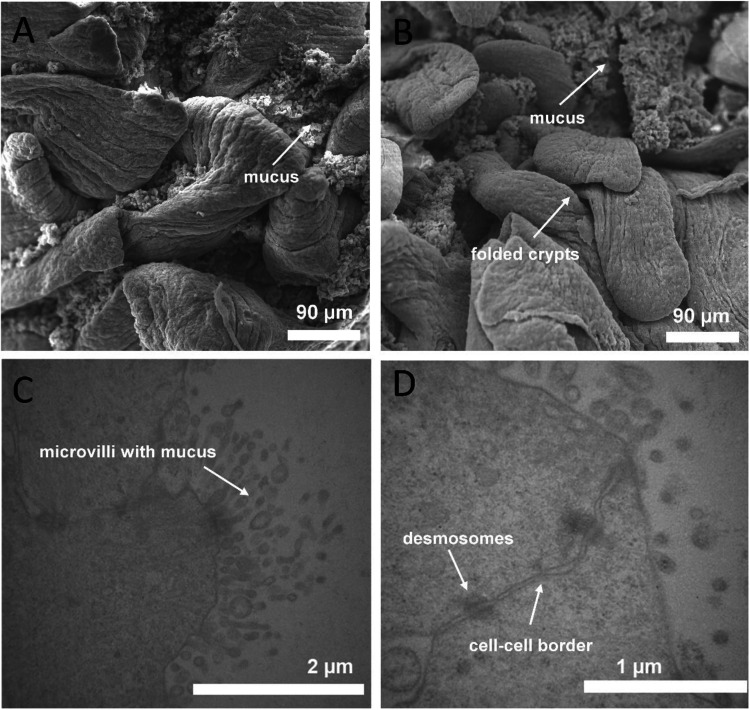
Electron microscopic characterization of small intestinal tissue from *Sus scrofa domestica.* **A**, **B** Scanning electron microscopy (SEM) indicates the folded crypts with overlying mucus structures. **C**, **D** Transmission electron microscopy (TEM) of embedded and sectioned tissue. **C** Microvilli and mucus. **D** Cell-cell-border with desmosomes, which connect both cells

Since there are many similarities between the physiology of the GIT in humans and pigs, porcine intestinal tissue offers a suitable alternative to non-primate and rodent animal models [37]. The fresh primary intestinal tissue used in this study was characterized via scanning electron microscopy (SEM) and transmission electron microscopy (TEM) to look for characteristics traits, which are typical of porcine intestine. SEM images identified undulated and folded crypts with overlying mucus structures (Fig. 2A, B), and TEM images clearly showed apical microvilli and mucus (Fig. 2C) and the cell-cell connection, with clearly visible desmosomes (Fig. 2D).

### Translocation of cellulose nanocrystals across primary porcine mucus

**Fig. 3:**
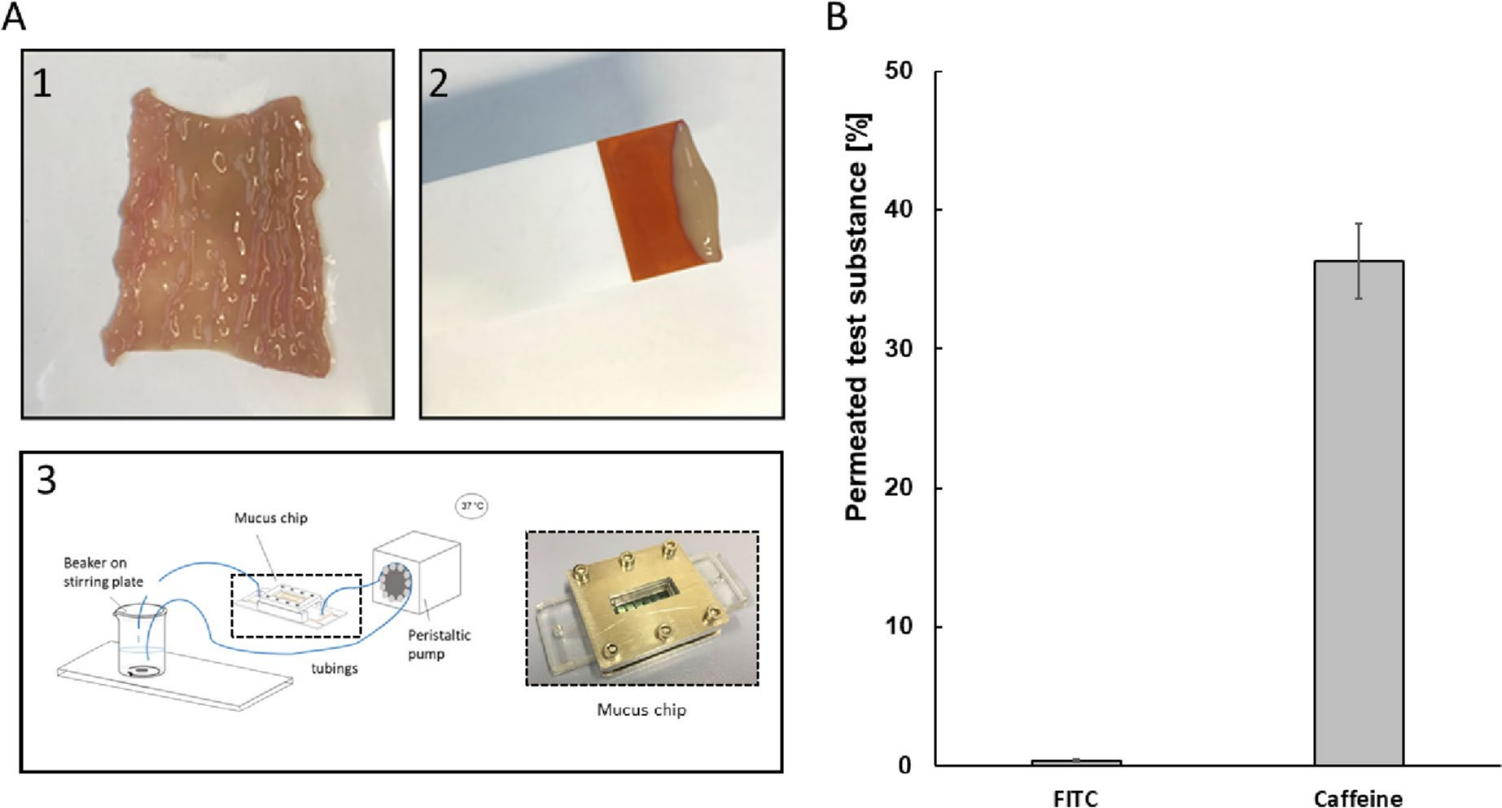
Translocation of test substances across primary porcine mucus. **A** Preparation of primary mucus and schematic test setup. 1: Primary intestinal tissue opened for mucus preparation. 2: Isolated primary mucus. 3: Schematic of technical setup of the microfluidic mucus chip system. **B** Permeation of FITC-dextran (100 μM, negative control) and caffeine (100 μM, positive control) was determined via HPLC and Tecan Infinite F200 plate reader (Tecan). Mean values present three independent experiments ± standard deviation

The microfluidic mucus chip system was used to investigate the ability of test compounds to overcome the mucus barrier. The test setup is shown schematically in Fig. 3A, consisting of a polystyrene-based microfluidic cartridge with polycarbonate membranes. Primary mucus was applied on the apical compartment of 200 μm thickness, to simulate the mucus layer in vivo. It acts as a barrier for substances before they reach the basolateral microfluidic channel, which is connected to a fluidic circuit, driven by a peristaltic pump (Cole Parmer). Caffeine was used as a positive control given its ability to readily pass the mucus barrier. FITC-dextran with a size of 70 kDa was used as negative control, since it is unable to pass the mesh structure of primary mucus [38]. After 2 h, 36.31 ± 2.72% caffeine and 0.38 ± 0.10% FITC-dextran permeated the mucus layer (Fig. 3B).

**Fig. 4:**
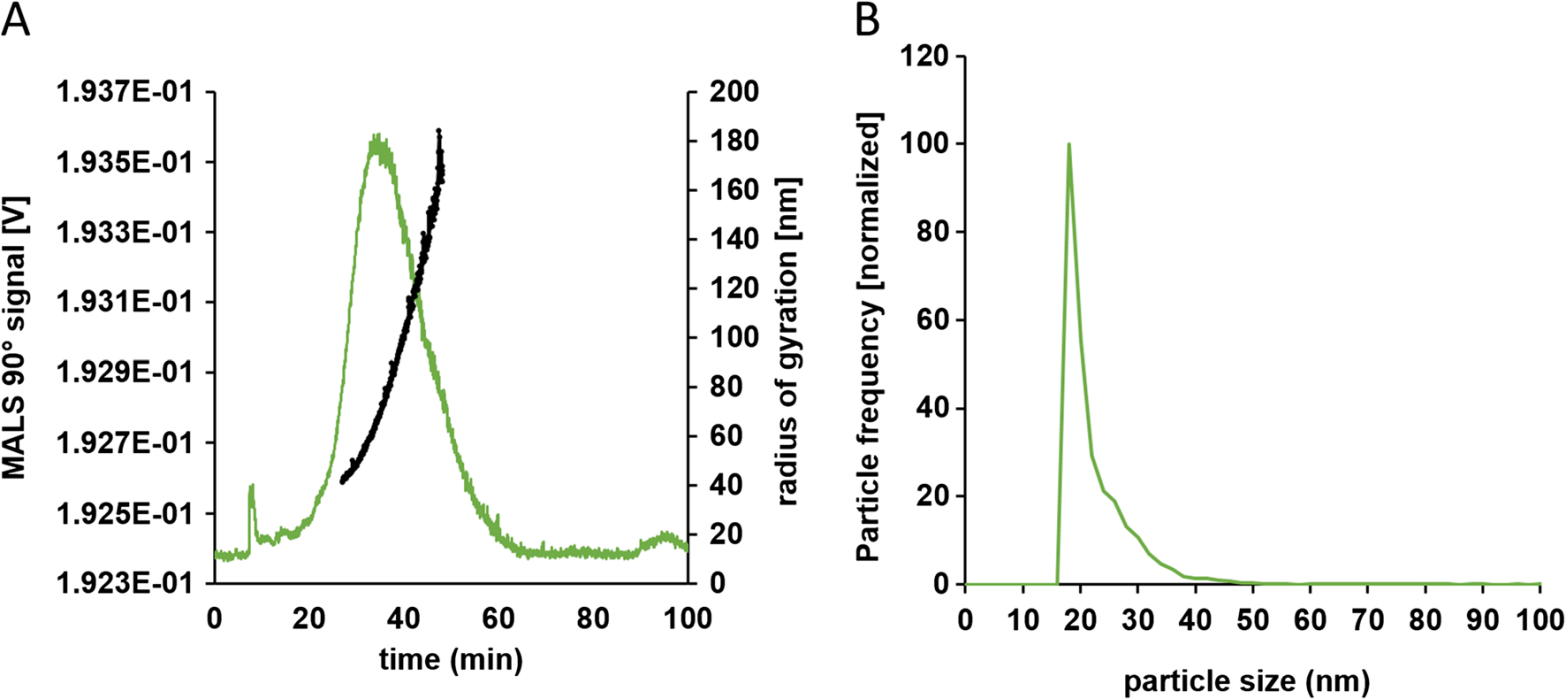
Analysis of basolateral samples for translocated cellulose nanocrystals and silver nanoparticles across intestinal mucus. **A** AF4-MALS fractogram of cellulose nanocrystals (green) with the radius of gyration (black) distribution. **B** Particle size distribution from spICP-MS analysis of silver nanoparticles (green). Diagrams exemplary for three runs and three biological replicates

After characterizing the microfluidic mucus chip model according to its barrier functionality, the transport of 100 μg·mL^−1^ CNC and 1 mg·mL^−1^ Ag-NP through the intestinal mucus was tested (Fig. 4). In all experiments, translocated CNC particles were detected on the basolateral side of the mucus. The size distribution is comparable to the CNC size distribution in KRB (before application to the mucus). In general, the heterogeneity of the samples caused differences between the studies. The R_g_ in the MALS 90° intensity maximum was calculated to around 67.8 ± 1.0 nm (Fig. 4A). In accordance with the results for translocated CNC, Ag-NP were also determined in basolateral samples. spICP-MS was able to detect a low degree of agglomeration (Fig. 4B). Moreover, the repeatability between studies and repeated measurements showed only low variations with a mean particle size of 21.3 ± 1.0 nm. Particles smaller than the size detection limit of around 15 nm could not be distinguished from the ionic silver concentration and were therefore counted to the ionic fraction.

### Translocation of cellulose nanocrystals across primary porcine tissue

**Fig. 5:**
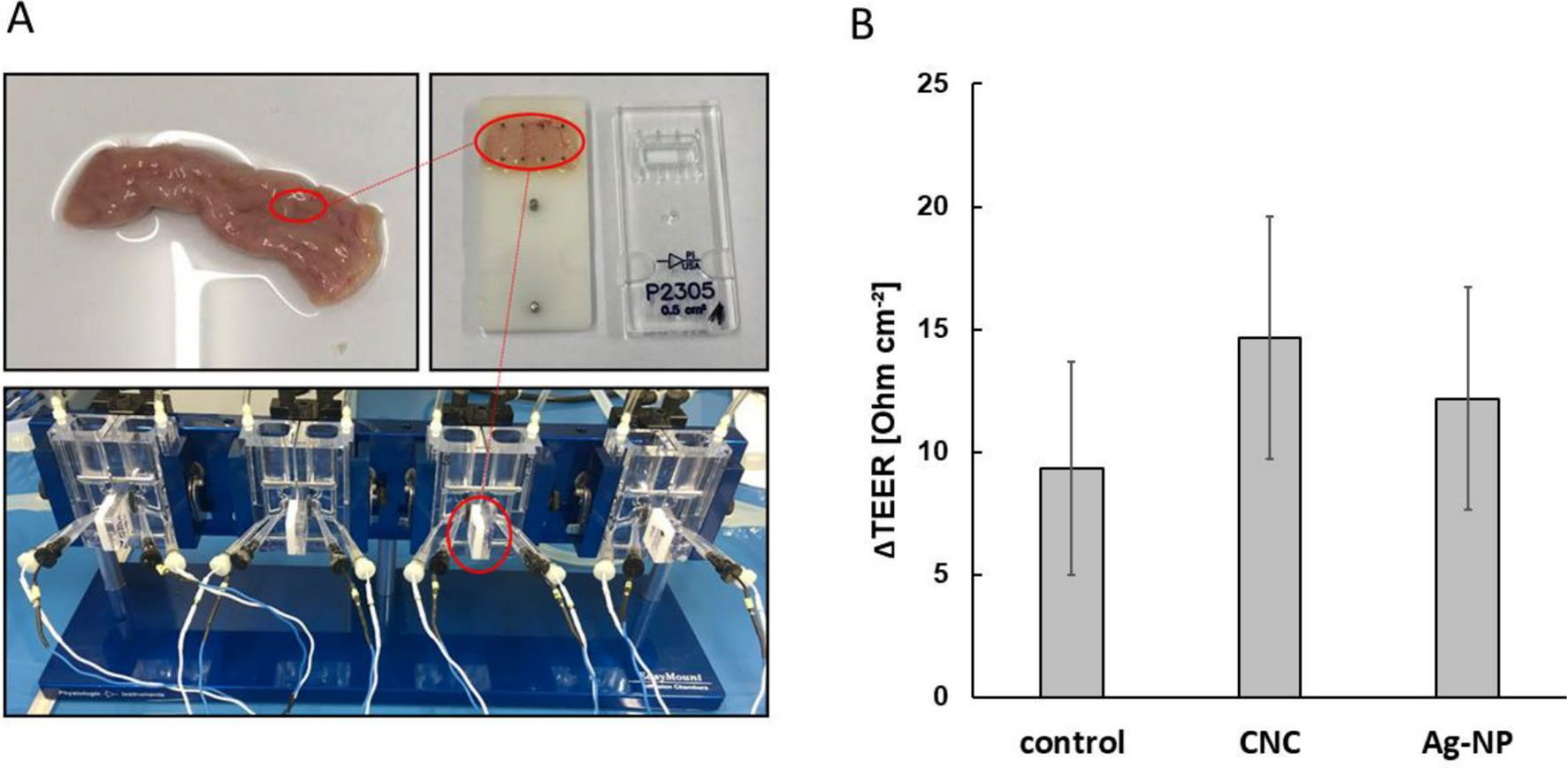
Preparation of porcine intestinal tissue and measurement of tissue integrity. **A** Preparation of tissue slices and setup in Ussing chamber. **B** Loss of barrier integrity (∆TEER) during 2 h apical exposure with 100 μg·mL^−1^ cellulose nanocrystals (CNC) or 1 mg·mL^−1^ silver nanoparticles (Ag-NP). Mean values of three independent experiments ± standard deviation

Since CNC were able to pass the mucus barrier representing the first hurdle of the intestinal absorption process, translocation studies were subsequently performed across primary intestinal tissue. The prepared tissue also includes a mucus layer by nature in addition to two more layers, the mucosa and submucosa, both of which have to be passed by the test compounds to reach blood vessels for a systemic distribution in the body. The prepared tissue and Ussing chamber apparatus are shown in Fig. 5A. The TEER has been measured during the whole test period of 2 h. ∆TEER is the decrease of the tissue resistance between the start of experiment and the end. Thus, the influence of the test nanomaterials, 100 μg·mL^−1^ CNC and 1 mg·mL^−1^ Ag-NP, on the barrier integrity is an indicator for toxicity and can be compared to an untreated intestine control. Because the treated tissue is artificially kept alive under ex vivo conditions by treatment with carbogen gas, serum-like bicarbonate buffer (KRB) and temperature control, a more pronounced loss of barrier integrity would be expected in the untreated control tissue. The results show that there was a small but not significant difference between the mean values of ∆TEER_(CNC)_ and ∆TEER_(Ag-NP)_ compared to ∆TEER_(Control)_ (Fig. 5B). After 2 h, samples were collected on the basolateral side for the analysis of translocated CNC and Ag-NP.

**Fig. 6:**
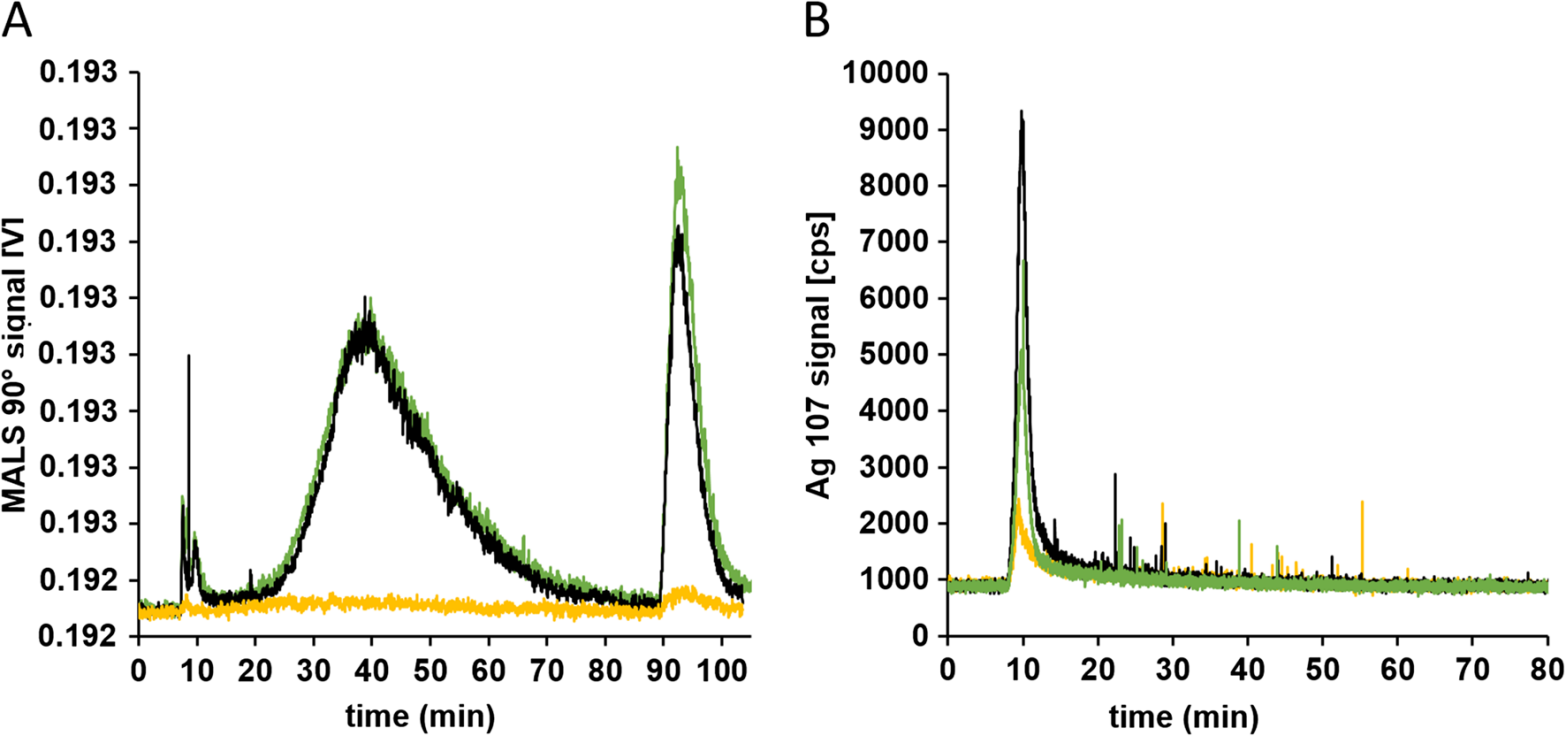
Analysis of basolateral samples for translocated cellulose nanocrystals and silver nanoparticles across intestinal tissue. **A** AF4-MALS fractogram of cellulose nanocrystals and blank measurements. **B** AF4-ICP-MS fractogram of silver nanoparticles. Yellow: KRB; black: control; green: basolateral sample. Diagrams exemplary for three runs and three biological replicates

Basolateral samples were fractionated and analyzed by AF4-MALS and AF4-ICP-MS, respectively. Firstly, the translocation of CNC particles across the intestinal tissue was investigated. After fractionation and subsequent analysis of the basolateral samples, no distinct fraction was detected, suggesting that there was no translocation or that it was immeasurable. Although the MALS signals indicated particulate fractions for some samples, these signals might originate from agglomerated proteins or other substances that were present in the samples rather than from translocated CNC particles, since the control samples showed identical peaks with the same height and width (Fig. 6A).

Secondly, the translocation potential for Ag-NP was investigated. All studies including Ag-NP revealed good repeatability. The ^107^Ag signal intensity fractogram of the basolateral samples was comparable to the control studies without the presence of particles. AF4-ICP-MS fractograms showed no translocation of particulate Ag-NP species above the size and concentration limit of the fractionation method (Fig. 6B). An increased void peak for the control and basolateral samples correlated with an increase in the peak for ionic silver. The overall intensities were low and varied slightly between measurements. Due to their high affinity to proteins of the cell culture medium, silver ions might also be removed from tubing and system components, contributing to the more pronounced void peak [39]. The fractionation method is able to resolve particles larger than around 5 nm from the void peak at around 8 min. Therefore, it can be concluded that no translocation of Ag-NP was observed above the detection limit of the detector (Fig. 6B).

### Comparison of ex vivo and in vitro model for application of transport and toxicity studies of cellulose nanocrystals

**Fig. 7:**
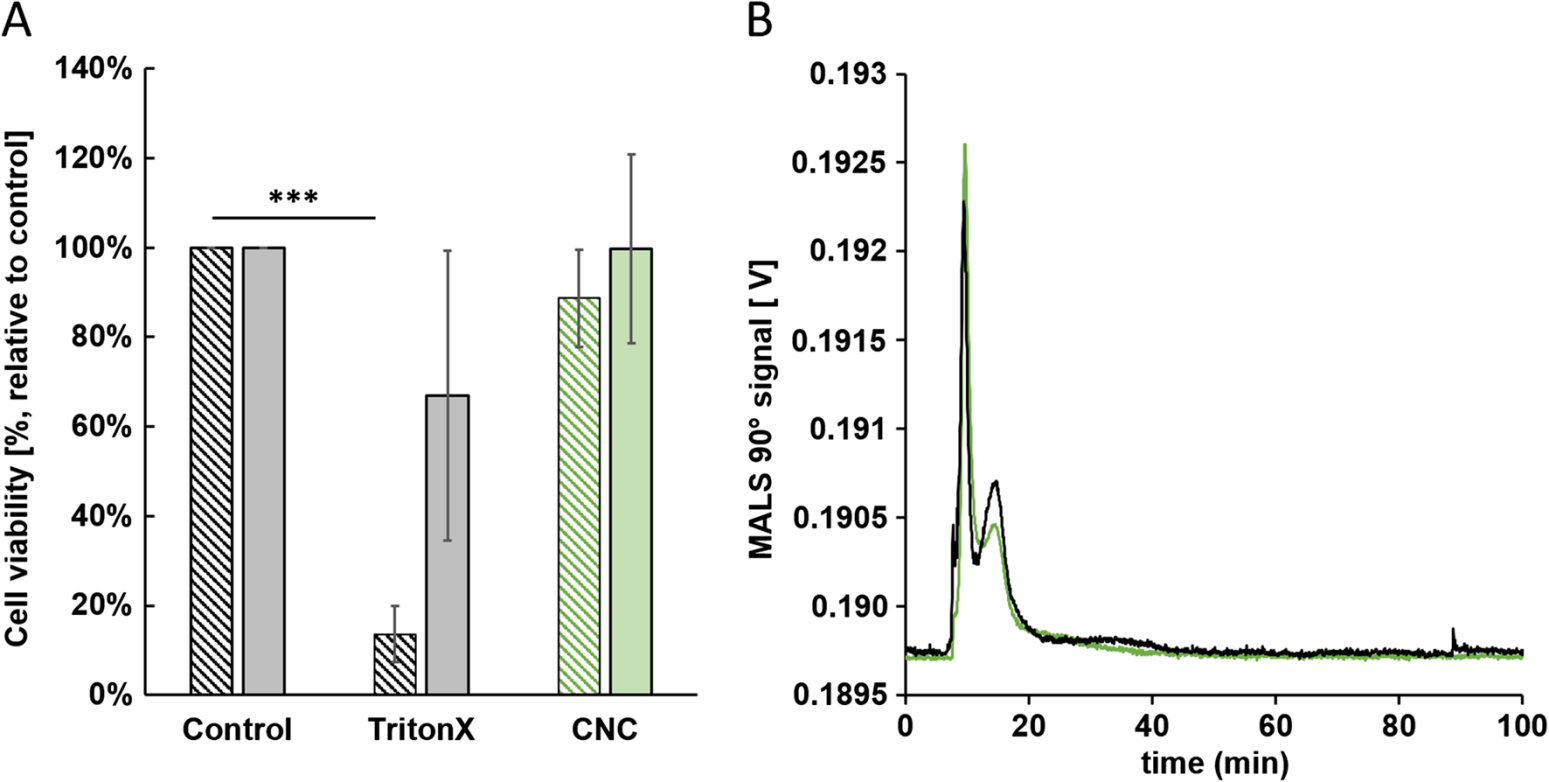
Translocation and cell viability study of cellulose nanocrystals in an in vitro triple co-culture model. **A** AF4-MALS fractogram of CNC in basolateral collected samples. Black: control; green: CNC. No translocated particles were detected in basolateral samples. **B** Cell viability of apical (lined bars) and basolateral (filled bars) cells after 2 h exposure to 100 μg·mL^−1^ CNC. Mean values of four independent experiments ± standard deviation. The difference between the mean value of control and TritonX group is considered as very significantly different, unpaired *t*-test (***p<0.0001)

An in vitro triple co-culture model was used to verify the results of the ex vivo with the in vitro experiments to provide insight into the accuracy of in vivo conditions. The in vitro model consists of three different cell types, absorptive enterocytes (Caco-2 cells), mucus-producing goblet cells (HT29-MTX-E12 cells), and monocyte-derived macrophages (PMA-differentiated THP-1 cells). The epithelial layer, which consists of 90% Caco-2 cells and 10% HT29-MTX-E12 cells according to the physiological ratio in vivo, was cultivated apically in this model. In vivo, macrophages are located in the lamina propria, which is directly found beneath the epithelial cell layer. Thus, macrophage-like cells differentiated from THP-1 cells were cultivated on the reversed Transwell^®^ membrane, directly beneath the epithelial cell layer. As it is in vivo, epithelial cells covered with a mucus layer stand in direct contact to ingested pathogens or nanomaterials. Macrophages can be activated to migrate into the mucosa upon external stimuli. Apical cells in this in vitro model were exposed to 100 μg·mL^−1^ CNC (same dose as in ex vivo). After 2 h exposure to basolateral samples, which were analyzed for translocated CNC fractions by AF4-MALS, no particles were transported, since the control samples showed identical peaks with the same height and width (Fig. 7A). The distinct void peak was caused by medium components. These results correlate to the data received in the ex vivo tissue model. In a second step, the epithelial cell viability (apical) and macrophage cell viability (basolateral) have been analyzed after 2 h. TritonX, which was used as membrane disrupting positive control, significantly decreased the apical cell viability from 100 to 13.58 ± 6.25% (Fig. 7B). Basolateral located cells were also affected, but this difference was not significant, assuming that the 21-day differentiated epithelial cell layer may act as a kind of protective shield for the basolateral cells. The viability of apical cells decreased slightly after exposure with CNC, whereas the activity of the basolateral cells was not affected. However, also these slight differences in comparison to the control were not significant (Fig. 7B). This result again correlates with the result obtained from the ex vivo tissue model, where no toxic effects on the intestinal tissue caused by CNC could be determined.

## Discussion

The 3R principle of Russell and Burch is a forward-thinking initiative. From an ethical and scientific standpoint, it should also be applied to in vivo experiments which demonstrate safety of drugs or new materials for humans. However, many existing in vitro models come with limitations regarding morphological and physiological features in comparison to the human intestine. Thus, they can only provide estimates of the absorption of drugs or other substances to the real in vivo situation [40]. In addition to the increasingly important in silico mathematical approaches such as Quantitative Structure-Activity Relationship (QSAR) modelling and Physiologically Based Kinetic and Dynamic (PBK/D) modelling, which aim to replace and reduce the use of animals in safety and efficacy testing, other approaches are also becoming more integral parts of safety assessment strategies. Integrated Approaches to Testing and Assessment (IATA) are flexible approaches to chemical safety assessment based on the integration and translation of data from different methods and sources and are effective in reducing animal testing. A multi-model approach, which uses ex vivo testing to address the physiological shortcomings of in vitro models, presents a promising solution. For this purpose, human tissue would be the most valuable source of explants for ex vivo experiments; however, healthy intestinal tissue is not usually available. This study demonstrates that by-products of the meat industry offer a suitable alternative owing to anatomic and physiological similarities of the porcine GIT with humans [41]. Length per body weight of the GIT is comparable in both species along with the epithelial cell population and the microbiota flora in small and large intestine [42]. Recent publications reported the use of porcine mucus for particle transport studies [41] and porcine intestine to study the permeability of drugs and dietary supplements [43, 44]. In the case of the safety assessment of nanomaterials, this approach considering the mucus and tissue barrier separately also offers a good opportunity to perform translocation studies and investigate mechanisms occurring in the gut epithelium following contact with new and emerging materials such as CNC.

Due to the growing demand for CNC-based products in the food industry, an increase in oral ingestion and exposure of the human GIT to these products is inevitable. It has been demonstrated that conventional cellulose is not absorbed from the GIT, but this could be different for nanosized cellulose such as CNC which has unique physicochemical properties [7]. There is a paucity of studies reporting on possible health effects or toxicity-related responses in the GIT upon oral uptake of CNC. Only in vivo [45–48] and in vitro studies [49–53] addressing this topic have been published recently. The investigation of the translocation of CNC across the intestinal barrier is especially under-reported [7].

Following oral uptake of compounds, the initial step of translocation into the blood vessels is passing the mucus barrier. With a net-like structure, mucosa acts as a steric barrier with filtering properties. Thus, the permeation of test compounds strongly depend on the size of its molecules [33], as demonstrated by the negative and positive controls investigated in this study. Caffeine is a small molecule (194.2 g·mol^−1^) and easily passed the mucus layer, whereas FITC with a size of 70.000 g·mol^−1^ is a very large molecule and was unable to cross the barrier [33, 54]. In this study, the integrity of the mucus layer is characterized by these control substances, because TEER measurements cannot be performed on mucus without an epithelium. Artificial mucus surrogates poorly simulate the structural characteristics and rheological properties of human mucus [36]. Therefore, primary mucus presents a much more suitable choice for permeation studies, which accurately mimic the human environment. A unique feature of the presented chip system is the ability to investigate the permeation of substances across the mucus layer alone, without the presence of epithelium. If substances are unable to pass the mucus layer, it is highly unlikely that the intestinal barrier will absorb them. One other recently published study investigated the transport of nanoparticulate drug carriers across a reconstituted mucus layer [55]. However, there are no other studies to date, which have examined the transport of unlabelled CNC solely across mucus. Lin et al. (2021) studied the influence of fluorescence-marked CNC on permeation through the porcine mucus but could not detect any CNC on the basolateral side. In contrast to the study described here, Lin et al. worked in a static system, investigated the CNCs in simulated intestinal fluid, and quantified CNC concentration by fluorescence spectroscopy. Lin et al. (2021) did not verify that the CNC are still in single particle state and not agglomerated in the intestinal fluid. In the present study, CNC were tested in KRB and CCM, exposed in a microfluidic system, and quantified via AF4-MALS. Due to the use of two different analysis methods based on different physical measurement principles that may result in different limits of quantification, the results of these two in vitro mucus translocation studies are not directly comparable.

To examine the ability of CNC to cross intact intestinal tissue, primary porcine intestinal tissue was characterized and used as an ex vivo model in which the intestinal epithelial integrity could be monitored. Since intestinal integrity is thought to be associated with diseases such as Crohn’s disease, ulcerative colitis, or colorectal cancer development, the effect of test substances on the barrier integrity is of great interest [56]. We have used the Ussing chamber system to provide valuable insight into intestinal integrity. Since neither the CNC nor the reference nanomaterial (Ag-NP) showed significant negative effects on the TEER in this study, it can be stated that these test compounds do not affect the intestinal epithelial integrity. Moreover, they did not cross the primary tissue from the mucosal side to the serosal side. To the best of our knowledge, this is the first study to investigate the translocation of CNC and Ag-NP in ex vivo models with primary tissues.

An in vitro triple co-culture model, which combines three of the most important abundant and influential cell types of the small intestine, was used to perform translocation studies that verify the ex vivo tissue models and further investigate toxic effects induced by CNC. Translocation studies using Ag-NP as reference material in the triple co-culture model have been described previously [34]. The exposure concentration and time were chosen according to those in the ex vivo models. Apical and basolateral viabilities were determined, and no loss of cell viability was induced by CNC neither in epithelial cells nor in the immune cells. This result supports the Ussing chamber experiments, in which the test nanomaterials also did not influence the integrity of the barrier. In agreement, a recent study on Caco-2 cells demonstrated that CNC exhibited low toxicity up to 10 mg·mL^−1^ test concentration [57]. This concentration is two orders of magnitude stronger than the one applied on the triple co-culture in vitro model and ex vivo intestine. However, conversely, another recent publication describes an increased cell membrane permeability and decreased cell metabolic activity of Caco-2 cells after 24 h of exposure to CNC at 50 μg·mL^−1^ [58]. While this is not in agreement with the in vitro triple co-culture and ex vivo intestine models, Caco-2 monolayers are greatly simplified models and may lack complex characteristics necessary to study CNC toxicity. However, regarding the translocation of CNC through epithelial layers, the study of Lin et al. (2021) is in line with the findings of this study. CNC could not translocate across any epithelial cell layers but were able to permeate the native mucus itself, suggesting that CNC may penetrate mucus barriers, but not epithelial linings.

The microfluidic mucus barrier model has given an insight into the translocation and transportation of CNC through the intestinal mucosa and tissue. Together with an in vitro model and Ussing chamber, this study has shown that CNC can translocate through intestinal mucus but not through primary intestinal tissue, and when CNC reaches the intestinal tissue, it does not have any toxic effect on the cell structures.

The mucus barrier model allows for rapid assessment of whether a substance can cross the mucus barrier, presenting a useful tool to inform the type and complexity of secondary test strategies for the absorption of compounds. The authors recommend that the mucus barrier model should be considered alongside a battery of other test methods for use with uptake studies. Used alongside ex vivo experiments and in vitro models in a multi-faceted approach, relevant insight can be gained into the complex physiological and toxicological interaction of new materials with the human intestine, during chemical and drug safety assessment. In addition to being cost-effective and providing a more accurate prediction of human interactions than in vitro techniques alone, this new approach also brings ethical advantages over animal studies. Furthermore, it aligns with IATA and NAMs for chemical and drug safety assessment based on the integration and translation of data from different methods.

## Supplementary information

ESM 1 (DOCX 490 kb)

## Data Availability

Supporting information is provided separately with the article. The datasets used and analyzed during the current study are available from the corresponding author on reasonable request.
