# Supplementary material for: Ex vivo models for intestinal translocation studies of cellulose nanocrystals
Source: In Vitro Model. 2023 Aug 21;2(5):181–94. doi: 10.1007/s44164-023-00056-x (PMC11756450; doi:10.1007/s44164-023-00056-x)
Supplement: Supplementary file 1 — (DOCX 490 kb) [file 44164_2023_56_MOESM1_ESM.docx]

***Ex vivo* models for intestinal translocation studies of cellulose nanocrystals**

Michelle Müller^1^, Roland Drexel^2^, Marie Burkhart^1^, Stephan Dähnhardt-Pfeiffer^3^, Lena Wien^1^, Christine Herrmann^4^, Thorsten Knoll^1^, Christoph Metzger^4^, Heiko Briesen^4^, Sylvia Wagner^1^, Florian Meier^2^ and Yvonne Kohl^1#^

^1^ Fraunhofer Institute for Biomedical Engineering IBMT, Department Bioprocessing & Bioanalytics, Joseph-von-Fraunhofer-Weg 1, 66280 Sulzbach/Saar, Germany

^2^ Postnova Analytics GmbH, Rankinestr. 1, 86899 Landsberg am Lech, Germany

^3^ Microscopy Services Dähnhardt GmbH, Plambeckskamp 2, 24220 Flintbek, Germany

^4^ Process Systems Engineering, School of Life Sciences, Technical University Munich, Gregor-Mendel-Str. 4, 85354 Freising, Germany

^#^ Corresponding author: Dr. Yvonne Kohl, yvonne.kohl@ibmt.fraunhofer.de, phone: +49 6897 9071256

**Supplementary Information**

**
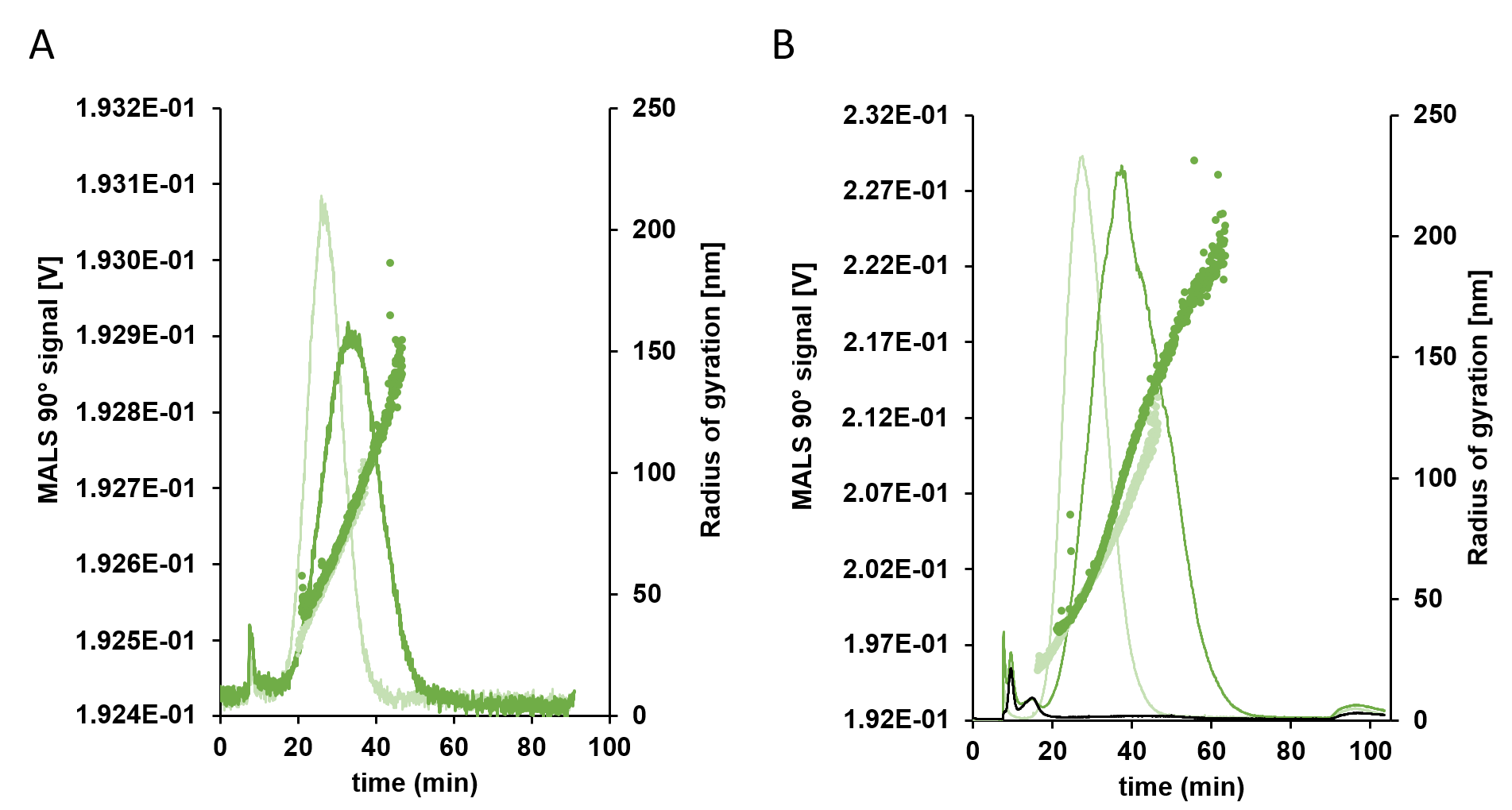
**

**Fig. S1: Characterization of cellulose nanocrystals in water, Krebs-Ringer buffer and cell culture medium. A:** AF4-MALS fractogram of cellulose nanocrystals (CNC) with the radius of gyration distribution**.** Light green: CNC in water; dark green: CNC in Krebs-Ringer buffer. **B:** AF4-MALS fractogram of CNC with the radius of gyration distribution. Black: cell culture medium; Light green: CNC in water; dark green: CNC in cell culture medium. Diagrams show one of three performed runs.

**
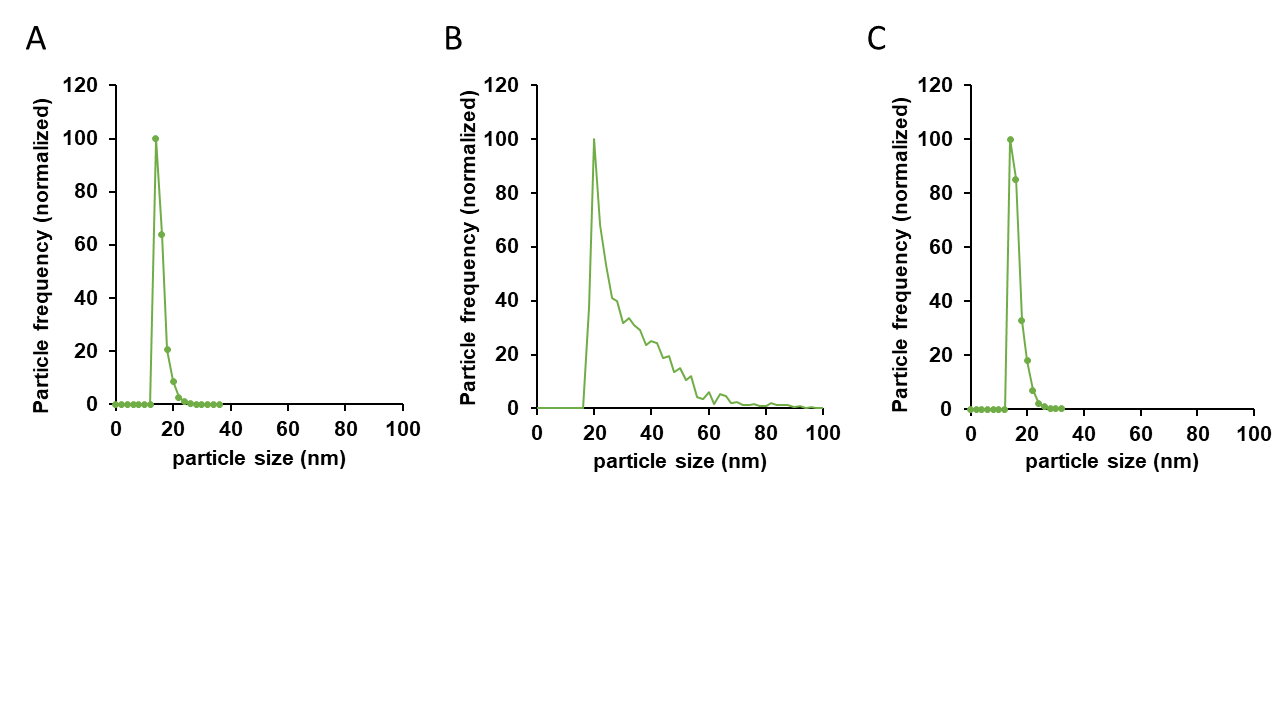
**

**Fig. S2: Characterization of silver nanoparticles in water, Krebs-Ringer buffer and cell culture medium with single particle ICP-MS analysis. A:** Particle size distribution of silver nanoparticles in water. **B:** Particle size distribution of silver nanoparticles in Krebs-Ringer buffer. **C:** Particle size distribution of silver nanoparticles in cell culture medium. Diagrams show one of three performed runs.
